# Ginsenoside Rg2 Ameliorates Myocardial Ischemia/Reperfusion Injury by Regulating TAK1 to Inhibit Necroptosis

**DOI:** 10.3389/fcvm.2022.824657

**Published:** 2022-03-22

**Authors:** Yao Li, Hao Hao, Haozhen Yu, Lu Yu, Heng Ma, Haitao Zhang

**Affiliations:** ^1^Clinical Medical College of Air Force, Anhui Medical University, Hefei, China; ^2^Department of Pathology and Pathophysiology, School of Basic Medical Sciences, Fourth Military Medical University, Xi’an, China; ^3^School of Basic Medical Sciences, Shaanxi University of Chinese Medicine, Xianyang, China; ^4^Department of Pathology, Xijing Hospital, Fourth Military Medical University, Xi’an, China; ^5^Department of Cardiology, PLA Air Force Medical Center, Beijing, China

**Keywords:** myocardial ischemia/reperfusion, necroptosis, ginsenoside Rg2, transforming growth factor β activated kinase 1, cardioprotection and ischemia-reperfusion injury

## Abstract

Necroptosis contribute to the pathogenesis of myocardial ischemia/reperfusion (MI/R) injury. Ginsenoside Rg2 has been reported to have cardioprotective effects against MI/R injury; however, the underlying mechanism remains unclear. This work aimed to investigate the effect of ginsenoside Rg2 on necroptosis induced by MI/R and to explore the mechanism. In this study, hypoxia/reoxygenation (H/R) injury model was established in H9c2 cells. *In vivo*, male C57/BL6 mice were subjected to myocardial ischemia 30 min/reperfusion 4 h. Rg2 (50 mg/kg) or vehicle was intravenously infused 5 min before reperfusion. Cardiac function and the signaling pathway involved in necroptosis were investigated. Compared with H/R group, Rg2 significantly inhibited H/R-induced cardiomyocyte death. Rg2 treatment effectively inhibited the phosphorylation of RIP1, RIP3, and MLKL in H/R cardiomyocytes, and inhibited RIP1/RIP3 complex (necrosome) formation. In mice, Rg2 treatment manifested significantly lower ischemia/reperfusion (I/R)-induced myocardial necroptosis, as evidenced by decrease in phosphorylation of RIP1, RIP3, and MLKL, inhibited lactate dehydrogenase (LDH) release and Evans blue dye (EBD) penetration. Mechanically, an increased level of tumor necrosis factor α (TNFα), interleukin (IL)-1β, IL-6, and MCP-1 were found in MI/R hearts, and Rg2 treatment significantly inhibit the expression of these factors. We found that TNFα-induced phosphorylation of RIP1, RIP3, and MLKL was negatively correlated with transforming growth factor-activated kinase 1 (TAK1) phosphorylation, and inhibition of TAK1 phosphorylation led to necroptosis enhancement. More importantly, Rg2 treatment significantly increased TAK1 phosphorylation, enhanced TAK1 binding to RIP1 while inhibiting RIP1/RIP3 complex, ultimately reducing MI/R-induced necroptosis. These findings highlight a new mechanism of Rg2-induced cardioprotection: reducing the formation of RIP1/RIP3 necrosome by regulating TAK1 phosphorylation to block necroptosis induced by MI/R.

## Introduction

As terminally differentiated cells, cardiomyocytes have highly limited ability to regenerate. Excessive death of cardiomyocytes induced by injury stress and their pathological effects leads to a variety of cardiovascular diseases, such as myocardial infarction (MI), malignant arrhythmia, heart failure (HF), and sudden cardiac death (1). Ischemic heart disease (IHD) is the leading cause of death and disability worldwide (2). The best way to prevent myocardial ischemic injury is to restore myocardial blood flow, i.e., reperfusion. However, reperfusion elicits further damage to the heart, which is called myocardial ischemia/reperfusion (MI/R) injury. Therefore, elucidating the mechanism of cardiomyocyte death and determining the intervention measures are of great significance for the prevention and treatment of IHD.

In the past, most of the studies on cardiomyocyte death focused on apoptosis, which is usually considered as programmed cell death. Necrosis is initial considered to be an unregulated process. In 1988, studies pointed out that tumor necrosis factor α (TNFα) can trigger a kind of cell death with necrotic morphological features (3). With the discovery of necroptosis by Degterev et al. (4), death receptor stimulation under the condition of apoptosis defect (i.e., caspase inhibition) can still trigger cell death with morphological features of necrosis in some cell types, which supported the existence of programmed cell death and provided a new mechanism for the intervention of cell death. Necroptosis, a regulated form of necrosis, is mediated by death receptors such as tumor necrosis factor receptor 1(TNFR1), and is executed through the induction of the RIP1–RIP3 necroptotic complex (5). As a death receptor-mediated caspase-independent cell death model, necroptosis has further completed cell death mechanism theory. Meanwhile, necroptosis inhibitors showed significant preventive and therapeutic effects in a variety of stress injury models, indicating that blocking necroptosis may become a new strategy for the prevention and treatment of stress-related injuries. Necroptosis plays an important role in IHD. It has been reported that RIP3 knockout or the use of Necrostatin-1 (RIP1 inhibitor) can significantly improve MI/R injury, which confirmed that inhibition of necroptosis is an effective cardioprotection against MI/R injury (6, 7).

Ginsenosides are the main components of ginseng, which exert a variety of pharmacological effects, such as vasodilation, anti-tumor, anti-diabetes, anti-inflammation, anti-oxidation, and so on. Ginsenoside Rg2 is one of the compounds in the protopanaxatriol group (8). Studies have shown that Rg2 can significantly improve myocardial ischemia injury and reduce MI area by increasing myocardial oxygen utilization, enhancing superoxide dismutase (SOD), scavenging free radicals, and so on. Rg2 can increase the mRNA expression of endothelial nitric oxide synthase gene (eNOS), which is also studied in ginsenoside Rb1 and Re (9) protect cardiomyocytes, reduce the content of malondialdehyde (MDA), the metabolite of lipid peroxidation, and reduce MI (10). Previous studies have reported that ginsenoside Rg2 has a protective effect on hydrogen peroxide-induced cardiomyocyte injury and apoptosis in rats (11, 12). Rg2 with a variety of biological activities and pharmacological effects, has the potential value for the treatment of cardiovascular diseases. However, whether Rg2 can inhibit myocardial necroptosis during MI/R and the underlying mechanism remains unknown.

Transforming growth factor-activated kinase 1 (TAK1, also known as map3k7), as a node regulator of apoptosis and necrosis, plays an important role in regulating the formation of RIP1–FADD–Caspase8 and RIP1–RIP3 necrotic complex (13). Studies have shown that TAK1 phosphorylation is essential to the interaction of RIP1 and TAK1. At the same time, TAK1 phosphorylation can also block the formation of RIP1–FADD–Caspase8 cell death complex induced by TNF receptor (14). There is a potential regulatory relationship between TAK1 and RIP1, and RIP3 is involved in the necroptosis-related pathway. The relationship between Rg2, TAK1, and necroptosis in MI/R settings has never been determined *in vivo*.

Here, we demonstrated that Rg2 treatment promotes the phosphorylation of TAK1 and enhances its binding to RIP1, thereby inhibiting the RIP1/RIP3 complex formation, and ultimately preventing MI/R-induced necroptosis.

## Materials and Methods

### Animals

All animal experiments were approved by the Animal Ethical Experimentation Committee of the Fourth Military Medical University. Male C57BL/6 mice at age of 12 weeks weighting 22–25 g were used. All animals were breed with regular pellet diets *ad libitum* in conventional facility on conditions of 12:12-h light/dark cycle.

### Materials

Evans blue was purchased from Sigma-Aldrich (St. Louis, MO, United States). The antibody against RIP1 (3493), RIP3 (95702), MLKL (37705), phosphor-MLKL (91689), phosphor-RIP1 (31122), phosphor-TAK1 (9339S), TAK1 (5206S), and β-tubulin (15115) were obtained from Cell Signaling Technology (Beverly, MA, United States), phosphor-RIP3 (AF7443) were obtained from Affinity. TNF-α was purchased from Novoprotein and caveolin 3 (CaV3) (ab2912) was obtained from Abcam (Cambridge, MA, United States). The level of serum lactate dehydrogenase (LDH) was detected by the kit purchased from Genesource.

### Cells Culture and Treatment

The H9c2 cells were cultured in Dulbecco’s modified Eagle’s medium (DMEM) with 10% fetal bovine serum and 1% Penicillin–Streptomycin Solution. To establish the hypoxia-reoxygenation cell model *in vitro*, the cells were added DMEM without sugar and serum upon reaching 70–80% confluence, and then were placed in an anoxic box, and replaced the air in the anoxic chamber with 95% N_2_, 5% CO_2_ mixed gas until the oxygen concentration was less than 1%, and cultured in the incubator at 37°C for 9 h (15). After the hypoxia, time was reached; the cells were replaced with high glucose DMEM medium (complete medium) and treated with Rg2. Finally, the cells were cultured and incubate at 37°C 5% CO_2_, respectively, for 2 h.

### Cell Counting Kit-8 Assay

Cell viability was detected using a Cell Counting Kit-8 (CCK8) assay. H9c2 cells were cultured in 96-well plates at about 2 × 10^4^/well. After incubating in 5% CO_2_ incubator at 37°C for 1 h, CCK8 assay solution (10 μl) contained water-soluble tetrazolium salt (WST8) was then added to each well and the cells were incubated for 1 h. The optical density of each well was measured using a microplate reader at 450 nm.

### Detection of Lactate Dehydrogenase

Lactate dehydrogenase determination kit (rate method) produced by Shanghai Kean Science and Technology Company uses continuous monitoring method to determine the activity of LDH, liquid A and liquid B are mixed into working reagent according to the ratio of 1:5. *In vitro*, the supernatant culture medium 40 μl after H/R added with 200 μl working reagent was ready to detect relative LDH release. *In vivo*, blood samples were collected from control and MI/R (30 min ischemia/4 h reperfusion) mice subjected to vehicle or Rg2 and centrifuged for 10 min at 3,000 r.p.m. to obtain serum. The serum 2 μl plus 38 μl double distilled water and then the working reagent of 200 μl was added to detect.

### Establishment of Myocardial Ischemia/Reperfusion Model in Mice

The procedure of MI/R injury model was built as previously described (15). In short, animals were anesthetized with pentobarbital (65 mg/kg, i.p.). After tracheostomy, ventilation was sustained on the Harvard rodent respirator. A left thoracic incision was performed, and the left anterior descending coronary artery was blocked by placing a 7-0 silk suture slipknot. The slipknot was released after 30 min. Reperfusion was sustained for 4 h in acute ischemia/reperfusion (I/R) injury (for western blot analysis, immunohistochemistry). Electrocardiogram was connected to monitor ST-segment changes during ischemia period. Rg2 (50 mg/kg) or vehicle was administered to mice randomly *via* caudal tail injection 5 min before reperfusion (16), then obtained the heart and picked up the white infarct zone for western blotting.

### Myocardial Necroptosis Measurement

Mice were injected intraperitoneally Evans blue dye (EBD) dissolved in saline (10 mg/ml) 12 h before MI/R operation. The heart was excised to separate the ventricular myocardium, then embedded it in optimal cutting temperature (OCT) compound, and immediately froze it in liquid nitrogen, finally cut into 5 μm cryosections. The Cav-3 antibody was performed to label viable cardiomyocytes while EBD-labeled necroptosis as previously described (15). The image was pictured by a fluorescence microscope.

### Immunohistochemistry Analysis of TNFα

The heart was obtained in control, MI/R (30 min ischemia/2 h reperfusion) group and MI/R + Rg2 group, fixed in 4% paraformaldehyde for 24 h and embedded in paraffin. The tissue was cut into 5 μm slices, and incubated with TNF-α antibody overnight at 4°C, then the second antibody was incubated at 37°C for 1 h, finally observed under microscope.

### Echocardiographic Measurement and Infarct Size

Ejection fraction (EF) and fractional shortening (FS) were measured as previously described (15). To evaluate the extent of myocardial necrosis hearts were excised and stained 3 days after MI/R induction. Hearts were sectioned into 1-mm slices and imaged using a Leica microscope. Viable cardiac tissue in the ischemic area was red-stained with 2,3,5-triphenyltetrazolium (TTC) and myocardium in non-ischemic area was blue-stained with the Evan’s blue, and infarcted tissues were white or light yellow. The infarct size was calculated as infarct area divided by area at risk (IF/AAR) (17).

### Enzyme-Linked Immunosorbent Assay

Serum levels of TNFα were measured in control and MI/R (30 min ischemia/2 h reperfusion) mice subjected to vehicle or Rg2 according to instructions (Beyotime.PT512).

### Western Blot

Total protein was extracted quantitatively, and separated by 10% sodium dodecyl sulfate polyacrylamide gel electrophoresis (SDS-PAGE) (30 g/lane), then transferred to PVDF membrane. The membranes were blocked with 5% slim milk with TBST and incubated with the primary antibody at 4°C overnight, then washed with TBST and incubated with second antibody for 1 h at room temperature. β-tubulin was used as the loading control.

### Statistical Analysis

Quantitative data were analyzed by Prism 8.0, and were expressed as mean ± SEM. double-tailed unpaired Student’s *t*-test and one-way ANOVA with Turkey’s test correction were applied to analyze significance between two groups, statistical significance was considered at *p* < 0.05.

## Results

### Rg2 Inhibits Hypoxia/Reoxygenation-Induced Cardiomyocytes Death

**FIGURE 1:**
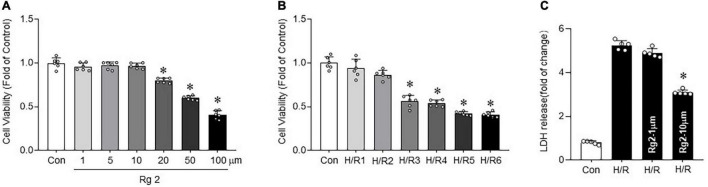
The Rg2 inhibits hypoxia/reoxygenation (H/R)-induced cardiomyocytes death. **(A)** The effect of gradient concentration of Rg2 (1, 5, 10, 20, 50, and 100 μM) on the viability of cardiomyocytes was detected by cell counting kit-8 (CCK8). **(B)** The effect of 1, 2, 3, 4, 5, and 6 h reoxygenation time after hypoxia 9 h on the viability of H9C2 cells was detected by CCK8. **(C)** Cell injury as assessed by LDH release after H/R (hypoxia 9 h and reoxygenation 3 h) with Rg2 treatment (1 and 10 μM). The values are the means ± SEM, *n* = 6 per group, *p < 0.05 vs. the control group.

By observing the effect of Rg2 concentration on cell survival rate by CCK8 assay, the results showed that Rg2 at a concentration of 1–10 μM has no effect on survival rate of H9c2, although an excessively high concentration of Rg2 (20–100 μM) will inhibit cell survival (Figure 1A). Next, to determine cell viability after H/R treatment, after hypoxia for 9 h, H9c2 cell viability was determined after various exposure times to reoxygenation time (1, 2, 3, 4, 5, and 6 h). The results showed that the cell survival rate decreased significantly at 3 h of reoxygenation (50.6 ± 2.5%) (Figure 1B). Therefore, a direct effect of Rg2 on H/R cardiomyocytes was assessed. H9c2 cardiomyocytes were treated with 1 and 10 μM Rg2 under hypoxia 9 h followed by reoxygenation 3 h. The results revealed that H/R treatment significantly increased H9c2 cell death evidenced by LDH release, while Rg2 administration significantly inhibited H/R-induced cardiomyocytes death in a dose-dependent manner (Figure 1C). In subsequent H/R experiments, hypoxia 9 h followed by reoxygenation 3 h is used. The results showed that Rg2 inhibits H/R-induced cardiomyocytes death.

### Rg2 Inhibits Hypoxia/Reoxygenation-Induced Cardiomyocytes Necroptosis

**FIGURE 2:**
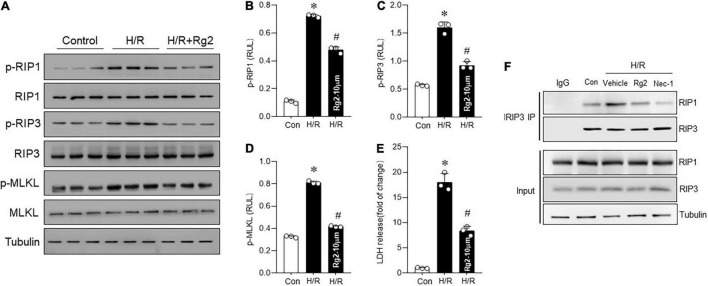
The Rg2 inhibits H/R-induced cardiomyocytes necroptosis. **(A)** Representative western blots in control, H/R group, and H/R + Rg2 group. **(B)** The averaged data for p-RIP1 level. **(C)** The averaged data for p-RIP3 level. **(D)** The averaged data for p-MLKL level. **(E)** Lactate dehydrogenase (LDH) release was detected to assess cardiomyocytes injury. **(F)** The Co-IP was conducted to detect the formation of RIP1/RIP3 necrosome. The values are the means ± SEM, *n* = 3 per group, *p < 0.05 vs. the control group, #*p* < 0.05 vs. the H/R group.

To determine whether there is a relationship between Rg2 and H/R-induced necroptosis in cardiomyocytes, RIP1, RIP3, and MLKL phosphorylation was determined. Compared with the control group, H/R significantly increased the RIP1, RIP3, and MLKL phosphorylation (Figures 2A–D) combined with increased LDH release (Figure 2E), suggesting activation of necroptotic pathway. Treatment with 10 μM Rg2 effectively inhibited H/R-induced necroptosis, as evidenced by reduced RIP1, RIP3, and MLKL phosphorylation and LDH release. Co-immunoprecipitation assays revealed that H/R-induced cardiac RIP1–RIP3 interaction was suppressed by Rg2 treatment (Figure 2F), which was similar to the effect of Nec-1 (a pharmacological inhibitor of RIP1 that blocks the RIP1–RIP3 interaction and inhibits necroptosis (18)). The above results confirmed that ginsenoside Rg2 can effectively inhibit H/R-induced cardiomyocytes necroptosis.

### Rg2 Attenuates Myocardial Ischemia/Reperfusion-Induced Necroptosis

**FIGURE 3:**
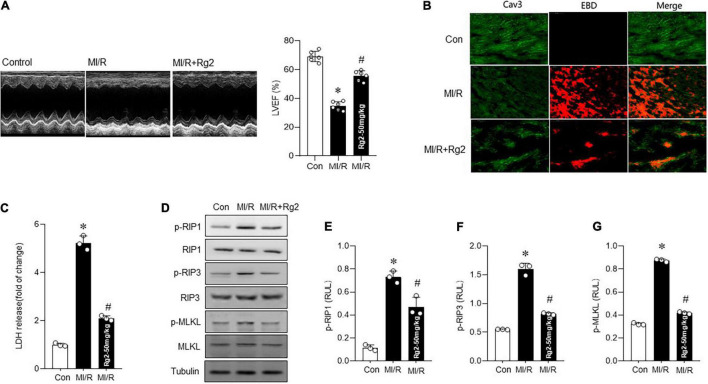
The Rg2 attenuates myocardial ischemia/reperfusion (MI/R) *in vivo* induced necroptosis. **(A)** Representative echocardiographic images in control and MI/R (30 min ischemia/4 weeks reperfusion) subjected to vehicle or Rg2. Left ventricular ejection fraction (EF) (LVEF)% was measured. **(B)** Representative photomicrographs from myocardial EBD uptake and viable cardiomyocytes labeled by caveolin 3 (CaV3) antibody in control, MI/R (30 min ischemia/4 h reperfusion), and MI/R + Rg2 group. **(C)** Myocardial injury as assessed by LDH release (30 min ischemia/4 h reperfusion). **(D–G)** Representative western blots in control, MI/R (30 min ischemia/4 h reperfusion) group and MI/R + Rg2 group and averaged data for p-RIP1, p-RIP3, and p-MLKL level. The values are the means ± SEM, *n* = 6 per group, *p < 0.05 vs. the control group, #*p* < 0.05 vs. the MI/R group.

To further explore the effect of Rg2 on MI/R injury *in vivo*, mice were subjected to 30 min ischemia followed by 4 h or 4 weeks reperfusion *in vivo* with vehicle or Rg2 treatment. The results demonstrated that Rg2 markedly reduced the I/R-induced myocardial infarct size to the area at risk, although the groups had comparable areas at risk (Supplementary Figure 1A). Furthermore, MI/R-induced (30 min ischemia/4 week reperfusion) cardiac contractile dysfunction (as indicated by decreases in EF) was rescued by Rg2 treatment (Figure 3A). We found that MI/R-induced (30 min ischemia/4 h reperfusion) cardiac necroptosis was markedly suppressed by Rg2 treatment, as evidenced by reductions in EBD penetration (Figure 3B) and LDH release (Figure 3C) in hearts. Accordingly, MI/R-induced myocardial RIP1, RIP3, and MLKL phosphorylation were effectively inhibited by Rg2 (Figures 3D–G). Thus, the *in vivo* data indicated that Rg2 attenuates MI/R-induced necroptosis.

### Rg2 Reduces TNFα and Ameliorates Myocardial Inflammation

**FIGURE 4:**
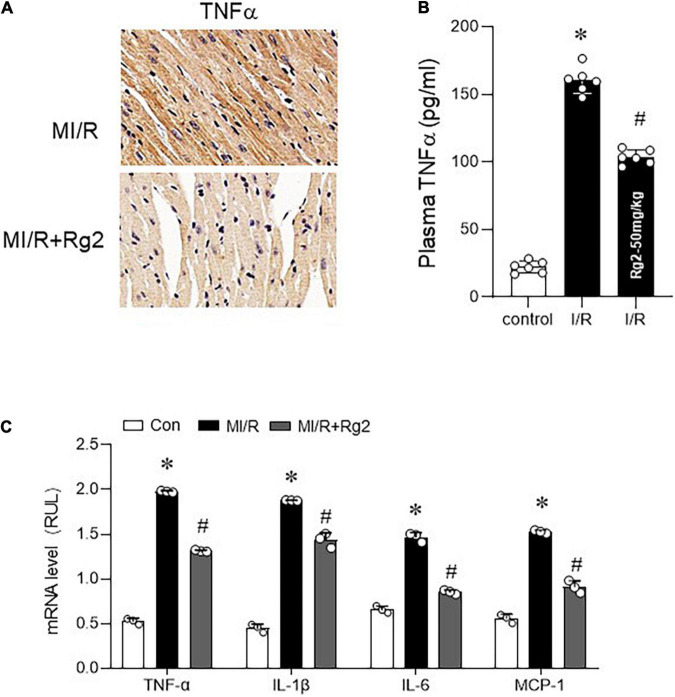
The Rg2 reduces tumor necrosis factor α (TNFα) and ameliorates myocardial inflammation. **(A)** Representative photomicrographs of myocardial TNFα (30 min ischemia/2 h reperfusion) demonstrated by IHC. **(B)** Plasma TNF-α concentration was evaluated after reperfusion for 2 h by ELISA assay. **(C)** The mRNA levels of TNFα, interleukin (IL)-1β, IL-6, and MCP-1 among control, MI/R, and MI/R + Rg2 group (30 min ischemia/2 h reperfusion). The values are the means ± SEM, *n* = 3 per group, *p < 0.05 vs. the control group, #*p* < 0.05 vs. the MI/R group.

Evidence shows that TNFα-induced necroptosis was involved in MI/R injury, myocardial TNFα level, and associated inflammatory factors were detected in MI/R (30 min ischemia/2 h reperfusion) myocardium. Myocardial TNFα level was markedly increased by MI/R injury, which was effectively mitigated by Rg2, as evidenced by enhanced TNFα IHC staining (Figure 4A), which is collaborated with plasma TNFα level (Figure 4B). Furthermore, Rg2 treatment significantly inhibited the MI/R induced increase in TNFα, IL-1 β, IL-6, and MCP-1 mRNA levels (Figure 4C), indicating that ginsenoside Rg2 can inhibit the activation of myocardial inflammation-related pathways induced by MI/R.

### Inhibition of Transforming Growth Factor-Activated Kinase 1 Phosphorylation Aggravates TNFα-Induced Necroptosis

**FIGURE 5:**
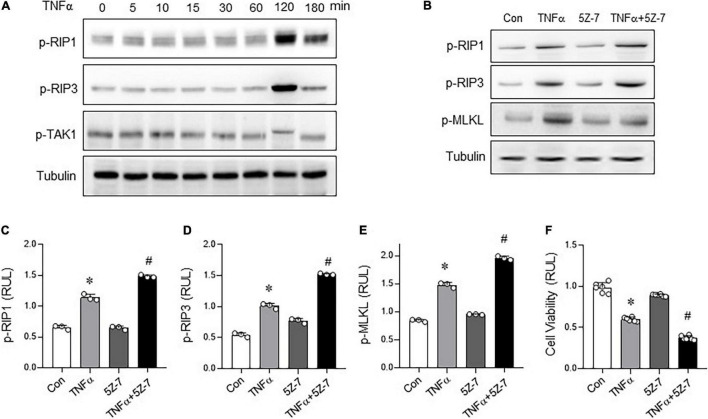
Inhibition of transforming growth factor activated kinase 1 (TAK1) phosphorylation aggravates TNFα-induced necroptosis. **(A)** Levels of p-RIP1, p-RIP3, and p-TAK1 in H9C2 cells with different treatment time of TNFα (10 ng/ml). **(B)** Levels of p-RIP1, p-RIP3, and p-MLKL in H9c2 cells treated with TNF-α (10 ng/ml, 2 h) with or without 5Z-7-OX (TAK1 inhibitor, 2 μM, pre-treatment 1 h) were detected by western blots. **(C–E)** p-RIP1, p-RIP3 and p-MLKL relative levels in H9c2 cells treated with TNF-α with or without 5Z-7-OX. **(F)** Cell viability was assessed by CCK8. The values are the means ± SEM, *n* = 3 or 6 per group, *p < 0.05 vs. the control group, #*p* < 0.05 vs. the TNFα group.

In order to further clarify how Rg2 regulates TNFα-induced necroptosis, H9c2 cardiomyocytes were stimulated by TNFα (10 ng/ml) to induce necroptosis. The results showed that 2 h after TNFα exposure, the RIP1 and RIP3 phosphorylation levels increased significantly (Figure 5A) associated with increased cell death (Figure 5F), while the phosphorylation of TAK1 decreased (Figure 5A), suggesting a negative relationship between the two. Furthermore, pre-treatment with 5Z-7-OX (2 μM, 1 h), a TAK1 phosphorylation inhibitor (19, 20), followed by TNFα exposure 2 h further enhanced the TNFα-induced RIP1, RIP3, and MLKL phosphorylation (Figures 5B–E) and cell death (Figure 5F). The above results confirm that inhibition of TAK1 phosphorylation further enhances TNF-induced necroptosis.

### Rg2 Enhances Transforming Growth Factor-Activated Kinase 1 Phosphorylation to Inhibit Necroptosis

**FIGURE 6:**
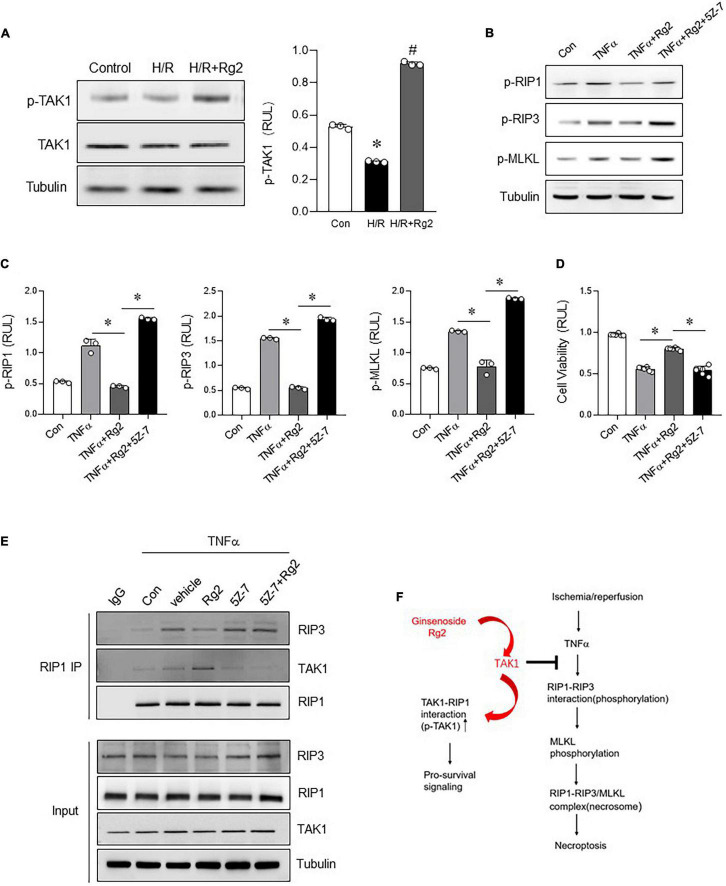
The Rg2 enhances TAK1 phosphorylation to inhibit necroptosis. **(A)** Levels of p-TAK1 and TAK1 in H/R with or without Rg2-treated H9c2 cells was detected by western blots. **(B)** Levels of p-RIP1, p-RIP3, and p-MLKL in H9c2 cells treated with TNF-α with or without Rg2 and 5Z-7-OX were detected by western blots. **(C)** Relative levels of p-RIP1, p-RIP3, and p-MLKL. **(D)** Cell viability was assessed by CCK8. **(E)** Co-IP was performed to detect the interaction between RIP1 and RIP3 or TAK1 in H9c2 cells treated with TNF-α with or without Rg2 and 5Z-7-OX. **(F)** Summary of this study. The values are the means ± SEM, *n* = 3 or 6 per group, *p < 0.05 vs. the TNFα or TNFα + Rg2 group.

Based on the above *in vitro* experiments, we further clarify whether Rg2 can regulate myocardial necroptosis through phosphorylation of TAK1. Indeed, TAK1 phosphorylation was markedly decreased in H/R injury H9c2 cells. However, compared with the H/R group, Rg2 effectively enhanced TAK1 phosphorylation (Figure 6A). In line with this notion, we also found that, in the TNFα-induced H9c2 necroptosis model, the protective effect of Rg2 can be blocked by 5Z-7-OX, as evidenced by enhanced RIP1, RIP3, MLKL phosphorylation and cell death (Figures 6B–D). More importantly, corresponding to the enhancement of TAK1 phosphorylation by Rg2, Co-IP results directly show that Rg2 treatment can enhance the binding ability of TAK with RIP1, and competitively inhibit the RIP1 and RIP3 binding to combine to form Necrosome (Figure 6E). The above data confirm that Rg2 inhibits myocardial Necroptosis by enhancing the phosphorylation of TAK1 (Figure 6F).

## Discussion

Our findings delineate that Rg2 treatment repressed myocardial necroptosis against MI/R injury both *in vitro* and *in vivo*. We have also identified Rg2 treatment effectively inhibited the inflammatory factors production in MI/R hearts, among which TNFα is an important factor in inducing necroptosis. The results of this study indicate that TAK1 phosphorylation leads to necroptosis inhibition. Rg2 enhanced TAK1 phosphorylation inhibits the RIP1 and RIP3 combination, and also inhibits myocardial necroptosis signal activation, and ultimately reduces MI/R injury. Our research provides new insights into the cardioprotective mechanism of Rg2.

In the past, most studies have focused on cardiomyocyte apoptosis, and it is believed that apoptosis is the only regulated form of cell death. However, necrosis, as the main pathological feature of various cardiac pathological conditions, has been completely ignored. Necrosis is considered to be an uncontrollable and passive mode of death. However, this view has since been challenged (21, 22). According to genetic and biochemical analysis, depending on the death initiating stimulus, necrosis is orchestrated and executed by appropriate mechanisms, rather than simply representing a disorganized breakdown of the cell. Nowadays, many studies have proved that necrosis also occurs in a regulated way and is closely related to the physiology and pathology in many organs, including the heart. As a regulated cell death form, necroptosis usually shows necrotic morphological characteristics depending on the interaction of RIP1, RIP3, and MLKL. Myocardial necroptosis can be activated by myocardial I/R, and the goal of treating myocardial I/R injury is to save ischemic myocardium, that is, to reduce myocardial cell death and reduce infarct size. Therefore, it is of important clinical and basic research significance to further clarify the potential mechanism of myocardial necroptosis in myocardial I/R injury and to find effective prevention and treatment strategies. Death receptor ligands such as TNF receptors can trigger a variety of cell responses, such as cell survival, apoptosis, and necrosis, depending on the type of cell stimulation (13). TNFR1 ligand binding leads to plasma membrane binding signal complex, called complex I, which consists of TNF receptor associated with death domain protein (TRADD), TNF receptor associated protein 2 (TRAF2), RIP1 and apoptosis inhibitor protein 1 and 2 (cIAP1 and cIAP2). Complex I recruits and activates the complex of TAK1 and I-receptor kinase (IKK), which leads to the activation of NFκB, which drives the transcription of pro-survival genes. Under certain conditions, complex I was disassociated from the membrane and transformed into an apoptosis-inducing complex, called complex II, and further recruited FADD (Fas-related protein and death domain) and caspase8. In addition, a complex composed of RIP1 and RIP3 (called necrosome) can be induced, which is very important for the initiation of necroptosis (23). Although some progress has been made recently, the precise molecular mechanisms that determine different cellular biological modes responding to TNFR1 have not been fully elucidated. In this study, it was found that Rg2 could inhibit myocardial necroptosis induced by TNF-α in myocardium subjected to I/R injury, promote the TAK1 phosphorylation and alleviate myocardial I/R injury.

Ginsenosides are the main components of Panax ginseng. Previous studies have shown that ginsenosides have myocardial protective effects. However, the potential mechanism of different types of ginsenosides on myocardial I/R injury remains unclear, which limits its clinical application. Ginsenoside Rg2 belongs to protopanaxatriol compounds. It has the effects of anti-tumor, anti-diabetes, anti-inflammation, anti-oxidation, and so on. Related studies have shown that Rg2 can inhibit cell apoptosis by activating SIRT1 and activating PI3K/AKT pathway, and has a protective effect on myocardial I/R injury in rats (11). However, about 70% of the cardiomyocytes death induced by H/R cannot be inhibited by zVAD (pan-caspase inhibitor), indicating that the cell death induced by H/R is mainly mediated by necrosis, and this kind of necrosis can be regulated (15, 24). However, it has not been reported whether ginsenoside Rg2 has regulatory effect on necroptosis, the dominant cell death in myocardium subjected to I/R. The study confirmed that ginsenoside Rg2 had obvious inhibitory effect on MI/R-induced necroptosis both *in vivo* and *in vitro*, and could attenuate myocardial inflammation, which further clarified the myocardial protective mechanism of Rg2 and provided a theoretical basis for the clinical application of Rg2.

Transforming growth factor activated kinase 1 is essential for regulating many important biological processes, such as immune cell activation, inflammation, cell differentiation, and cardiac hypertrophy. Previous studies have reported the role of TAK1 in regulating cardiomyocyte apoptosis and necroptosis (25). TAK1 regulates and maintains myocardial homeostasis and prevents cardiac remodeling by controlling cardiac programmed cell death (26, 27). Cardiac specific knockout of TAK1 induces spontaneous apoptosis and necroptosis of cardiomyocytes, followed by poor remodeling and heart failure (13). The related studies have reported that TAK1 plays a role in cardiotoxicity induced by DOX. The expression of TAK1 is decreased in cardiotoxicity induced by DOX *in vivo* and *in vitro*, and TAK1 inhibition by TAK1 phosphorylation inhibitor 5Z-7-OX would further aggravate cardiomyocyte apoptosis and necroptosis induced by DOX (28). TAK1 phosphorylation is very important for its catalytic activity (29). Previous studies have shown that the inactivation of TAK1 in cardiomyocytes eliminates the phosphorylation of c-Jun-N-terminal kinase (JNK) induced by TNFα and the degradation of IKκB, thus inhibiting the activation of JNK and NFκB pathway, indicating that TAK1 phosphorylation is essential for the activation of pro-survival pathway induced by TNFα (13). And the study initially targeting TAK1 phosphorylation was performed on non-alcoholic fatty liver disease (NAFLD) (30). At present, related studies have revealed the biological role of TAK1 in regulating myocardial survival/death and cardiac homeostasis. However, the protective mechanism of TAK1 has not been determined yet. In this study, it confirmed that there was an antagonistic relationship between TNFα-induced necroptosis and TAK1 phosphorylation. Rg2 could play a cardioprotective role *via* enhancing the phosphorylation of TAK1, and promote the interaction between TAK1 and RIP1. Thus, Rg2 prevents the formation of RIP1/RIP3 necrosome inhibiting necroptosis. This study suggests a new mechanism of cardioprotection for Rg2 and further reveals the role of TAK1 phosphorylation in regulating TNFα-induced myocardial necroptosis. However, whether Rg2 promotes TAK1 phosphorylation directly or indirectly to inhibit necroptosis needs to be further studied. In addition to TAK1 phosphorylation, whether it is related to other modifications, such as ubiquitin, TAK1 as a molecular switch of programmed cell death, whether the activation time of TAK1 phosphorylation will alter its inhibitory effect on necroptosis, such as whether hyperphosphorylation will reverse the inhibitory effect of necroptosis. These problems need to be further discussed. Some studies have shown that regulator of G protein signaling 5 (RGS5) can directly interact with TAK1 to inhibit its hyperphosphorylation and thus inhibit c-JNK/P38 signaling pathway, thus effectively alleviating the progression of NAFLD (31). Meanwhile, RGS5 can inhibit cardiomyocyte apoptosis in myocardial I/R injury (30). However, whether the protective mechanism of Rg2 in I/R myocardial necroptosis induced by TNFα *via* regulating TAK1 is related to the activation of RGS5 and the interaction between RIP1-TAK1-RGS5 remains to be further elucidated. In addition, due to the limitations of the H9c2 cell line, more *in vitro* experiments are needed to determine the generality of our findings, such as using the human-induced Pluripotent Stem Cell derived cardiomyocytes (hiPSC-CMs).

## Conclusion

In conclusion, based on *in vivo* and *in vitro* experiments, the study demonstrates that TAK1 phosphorylation plays an important role in regulating myocardial necroptosis induced by TNFα. Rg2 enhances TAK1 phosphorylation to inhibit necroptosis. This research is important in furthering our understanding of Ginsenoside-induced cardioprotection.

## Data Availability Statement

The original contributions presented in the study are included in the article/Supplementary Material, further inquiries can be directed to the corresponding authors.

## Ethics Statement

The animal study was reviewed and approved by the Animal Ethical Experimentation Committee of the Fourth Military Medical University. Written informed consent was obtained from the owners for the participation of their animals in this study.

## Author Contributions

YL, HY, and HH performed the material preparation, data collection, and analysis. LY, HM, and HZ wrote the first draft of the manuscript. All authors commented on previous versions of the manuscript, contributed to the study conception and design, and read and approved the final manuscript.

## Conflict of Interest

The authors declare that the research was conducted in the absence of any commercial or financial relationships that could be construed as a potential conflict of interest.

## Publisher’s Note

All claims expressed in this article are solely those of the authors and do not necessarily represent those of their affiliated organizations, or those of the publisher, the editors and the reviewers. Any product that may be evaluated in this article, or claim that may be made by its manufacturer, is not guaranteed or endorsed by the publisher.
