# Supplementary material for: Ginsenoside Rg2 Ameliorates Myocardial Ischemia/Reperfusion Injury by Regulating TAK1 to Inhibit Necroptosis
Source: Front Cardiovasc Med. 2022 Mar 22;9:824657. doi: 10.3389/fcvm.2022.824657 (PMC8981204; doi:10.3389/fcvm.2022.824657)
Supplement: Supplementary file 1 [file Data_Sheet_1.pdf]

Supplementary Data

Ginsenoside Rg2 ameliorates myocardial ischemia/reperfusion injury by regulating TAK1 to inhibit necroptosis

Yao Li [1], Hao Hao[2], Haozhen Yu[3], Lu Yu[4, #], Heng Ma[2,#], Haitao Zhang[1,5,#]

[1] Clinical Medical College of Air Force, Anhui Medical University, Hefei 230032, China;

[2] Department of Pathology and Pathophysiology, School of Basic Medical Sciences; Fourth Military Medical University, Xi'an 710032, China;

[3] Shaanxi University of Chinese Medicine, Xianyang 712046, China;

[4] Department of Pathology, Xijing Hospital, Fourth Military Medical University, Xi'an 710032, China;

[5] Department of Cardiology, Air Force Medical Center, PLA, Beijing 100142, China

Short Title: Ginsenoside Rg2 inhibits myocardial necroptosis

Word count: 4800

Figures: 6

Correspondence to:

Haitao Zhang, M.D., Ph.D.

1.Clinical Medical College of Air Force, Anhui Medical University, Hefei 230032, China; 2.Department of Cardiology, Air Force Medical Center, PLA, Beijing 100142, China. E-mail: kjzht@sina.com

Heng Ma, M.D., Ph.D.

Department of physiology and pathophysiology, School of Basic Medical Sciences, Fourth Military Medical University, Xi'an 710032, China. E-mail: hengma@fmmu.edu.cn

Lu Yu, M.D., Ph.D.

Department of Pathology, Xijing Hospital, Fourth Military Medical University, Xi'an 710032, China. E-mail: yulu@fmmu.edu.cn

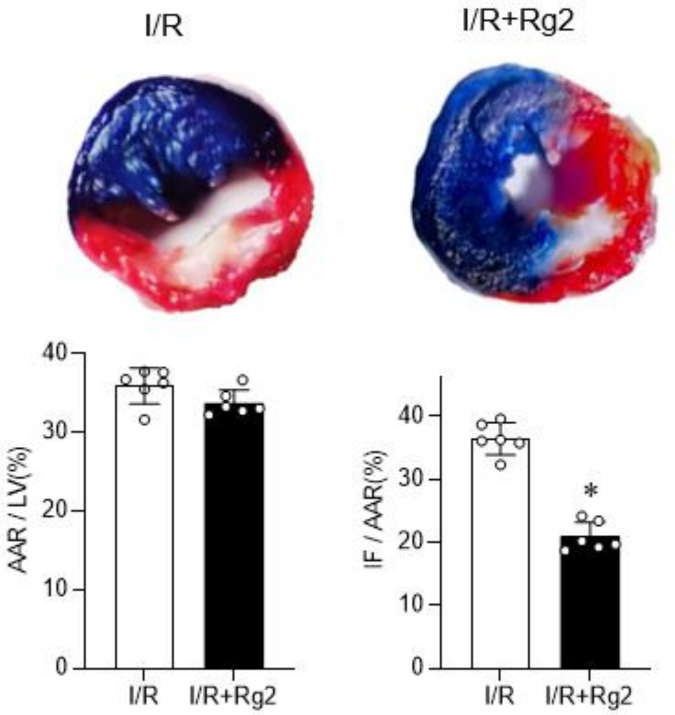

Supplementary Figure 1. a. Representative photographs and quantitative data for infarct size (IF) and area at risk (AAR) in hearts from mice subjected to MI/R with vehicle or Rg2. b. Representative echocardiographic images. Left ventricular ejection fraction (LVEF)% and LV fractional shortening (LVFS)% was measured. The values are the means ± SEM, n=6 per group, ****P*<0.05 versus the control group, # *P*<0.05 versus the I/R group.**

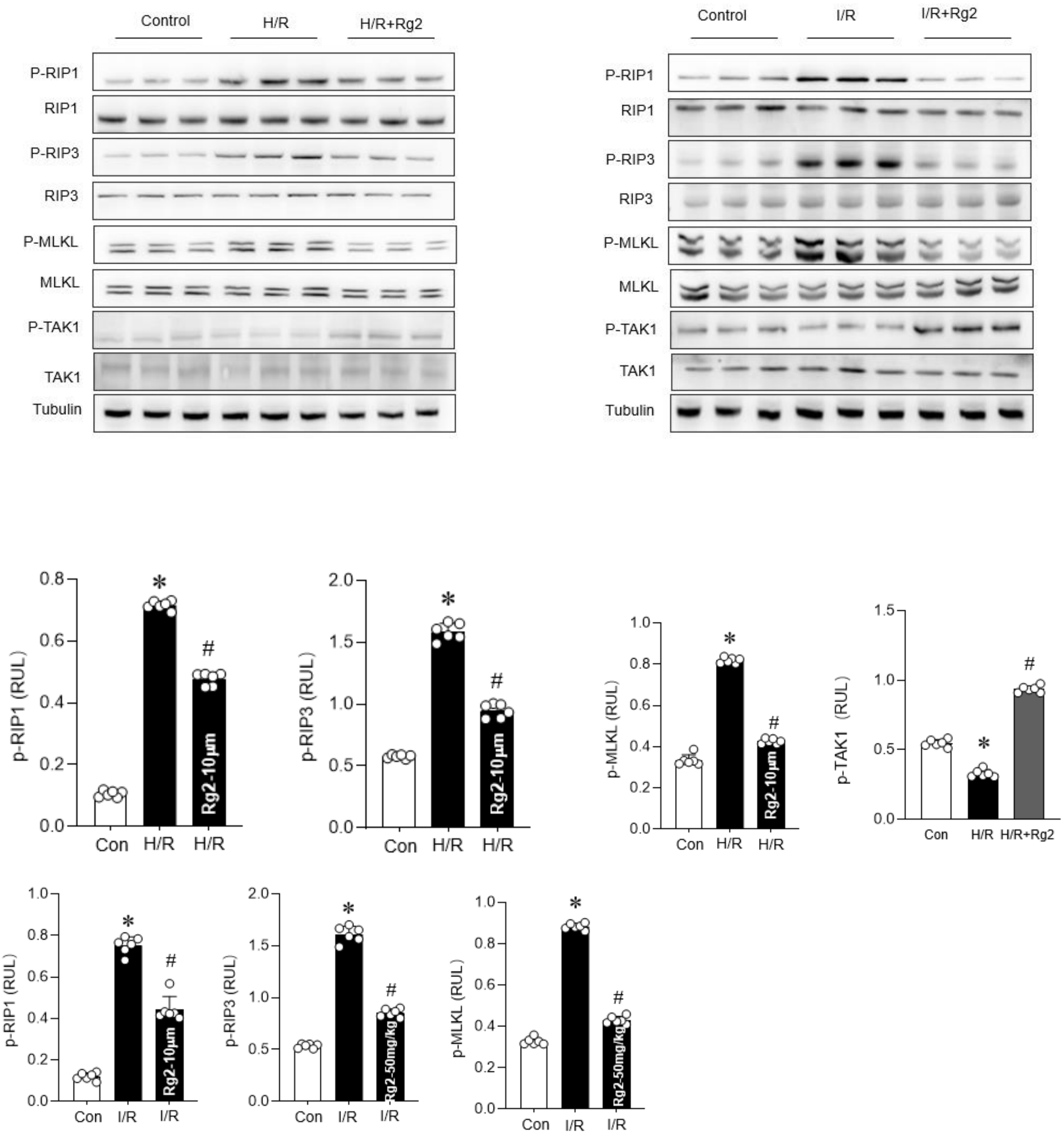

Supplementary Figure 2. Quantitated p-RIP1, RIP1, p-RIP3, RIP3, p-MLKL, MLKL, p-TAK1 and TAK1 both in vitro and in vivo. The values are the means ± SEM, n=6 per group, ****P*<0.05 versus the control group, # *P*<0.05 versus the I/R group.
